# Prevalence of Methicillin-Resistant *Staphylococcus aureus* in Livestock in Japan: A Systematic Review and Meta-Analysis

**DOI:** 10.3390/epidemiologia6010003

**Published:** 2025-01-24

**Authors:** Sayoko Hanamoto, Yuri Fujimoto, Katsuaki Sugiura, Takeshi Haga

**Affiliations:** 1 Division of Infection Control and Disease Prevention, Department of Veterinary Medical Science, Graduate School of Agricultural and Life Sciences, The University of Tokyo, Tokyo 113-8657, Japan; hanamoto-sayoko0908@g.ecc.u-tokyo.ac.jp; 2 Laboratory of OSG Veterinary Science for Global Disease Management, Graduate School of Agricultural and Life Sciences, The University of Tokyo, Tokyo 113-8657, Japan; 3 Laboratory of Environmental Science for Sustainable Development, Graduate School of Agriculture and Life Sciences, The University of Tokyo, Tokyo 113-8657, Japan; aksugiur@g.ecc.u-tokyo.ac.jp; 4 Nippon Institute for Biological Science, Ome, Tokyo 198-0024, Japan

**Keywords:** methicillin-resistant *Staphylococcus aureus*, MRSA, animal, livestock, meta-analysis, prevalence

## Abstract

**Background:** Methicillin-resistant *Staphylococcus aureus* (MRSA) is an important health issue that is estimated to have caused 130,000 deaths worldwide in 2021. As more instances of cross-species transmission of MRSA have been reported, concerns have been raised regarding the spread of livestock-associated MRSA to humans. The prevalence of MRSA in livestock varies globally. This study systematically reviews the prevalence of MRSA at the farm and animal levels in Japan. **Methods:** Relevant studies published in English or Japanese between 2000 and 2023 were retrieved from four databases. Pooled prevalences at the farm and animal levels in Japanese farms were calculated using a random-effects model. Subgroup and meta-regression analyses were also performed to explore sources of heterogeneity. **Results:** The 13 studies included in this meta-analysis yielded an MRSA prevalence of 3.54% (95% confidence interval [CI] 0.65–8.30%) at the individual pig level, 13.07% (95% CI 5.42–23.04%) at the pig farm level, 0.0% (95% CI 0.00–0.04%) at the individual cattle level, and 0% (95% CI 0.00–0.44%) at the individual chicken level. A significant increase in MRSA prevalence over time was evident at the individual pig level by both subgroup analysis (*p* = 0.020) and meta-regression (*p* = 0.019). **Conclusions:** Our results indicated that the proportion of pigs that can be a source of MRSA infection in humans has been steadily increasing in Japan. Despite some limitations, our findings strongly imply a need for more attention to pig-to-human MRSA transmission in Japan.

## 1. Introduction

*Staphylococcus aureus* is a commensal bacterium that has adjusted well to human hosts [1]. Normally, it rarely causes symptoms in healthy individuals. However, it can lead to fatal infections depending on their immune status. Children, elderly people, and patients with immunosuppression are considered to be at high risk [2]. *S. aureus* is characterized by the rapid acquisition of resistance to most antibiotics used in clinical practice [3]. Methicillin-resistant *S. aureus* (MRSA) has gained resistance to methicillin via the acquisition of the *mecA* gene encoding penicillin-binding protein 2a (PBP2a), an enzyme to construct the bacterial cell wall targeted by β-lactams but having a low affinity for them [1]. In addition, MRSA confers resistance not only to most β-lactams including methicillin, but also to multiple other antibiotic classes [4]. There are known genes related to antimicrobial resistance of MRSA: *blaZ*, associated with penicillin resistance, *ermA/C*, associated with macrolide resistance, and *tetK/M*, associated with tetracycline resistance [1].

Once MRSA enters the host body, it may cause pneumonia, bacteremia, endocarditis, osteomyelitis, and infections of the skin and soft tissues [1,3,5]. MRSA infection makes treatment much more difficult due to the limited antimicrobial options, thus increasing mortality rates. Global MRSA-attributable deaths have been estimated to have doubled from 57,200 in 1990 to 130,000 in 2021 [6]. In Japan, an estimated 4224 people died from MRSA bacteremia in 2017 [7], indicating that MRSA is a public health concern worldwide as well as in Japan. The prevalence of MRSA in the Asia Pacific region is considered comparable to those reported in Europe and the Middle East, whilst the prevalence has been indicated to be higher in East Asian countries compared with Southeast Asian countries [8,9]. Antibiotic consumption in middle- and low-income regions, including India and China, increased from 2000 to 2015 [10]. This trend could, possibly, contribute to the high mortality rates caused by antimicrobial resistance, represented by MRSA, among countries in South and Southeast Asia in 2050 [6].

MRSA can be classified based on clinical or molecular epidemiological investigations into hospital-associated (HA-MRSA), community-associated (CA-MRSA), and livestock-associated MRSA (LA-MRSA) [11]. The distinction between human-associated MRSA (comprising HA-MRSA and CA-MRSA) and LA-MRSA is unclear, as LA-MRSA has shown a potential to colonize both humans and animals [12]. The prevalence of MRSA is reportedly higher among people occupationally exposed to livestock [13]. On the other hand, students living in rural areas, rather than having livestock exposure, present a high prevalence of MRSA [14]. A dose–response relationship exists between the frequency and duration of exposure to livestock and the carriage rate of MRSA in humans [15]. Further, a positive association between livestock exposure and the carriage risk of LA-MRSA, clonal complex (CC)398 and CC9 has been reported [16]. CC398 and CC9 are considered the dominant LA-MRSA strains. The former has been detected in farms mostly in European and American countries, while the latter has been detected in farms mostly in Asian countries [17]. However, recent reports have found the emergence of CC398 from pig farms in China and South Korea [18,19]. Likewise, the isolation of the sequence type (ST)398, which belongs to CC398, from pigs has been reported in Japan [20,21,22,23], strongly implying the possibility of ST398 colonization among pigs in this country. Concern is growing regarding the transmission of LA-MRSA from livestock to humans, particularly to farmers, veterinarians, and slaughterhouse workers who are in close contact with the livestock.

The prevalence of MRSA in livestock varies by geographical region and animal species. A study conducted by the European Food Safety Authority showed that the prevalence of MRSA on pig farms varied widely from country to country, ranging from 0% to 46% [24]. A systematic review of MRSA prevalence in dairy farms found that prevalence varied from 4.89% in Asia to 1.33% in South America [25]. Recent cross-sectional research has revealed that the prevalence of MRSA in cattle nasal swab samples was 12.4% in India and 13.3% in Bangladesh, differing from pooled values [26,27]. Another systematic review of poultry revealed that the pooled prevalence of MRSA was highest in South America (27%), followed by Africa (16%), Europe (15%), Asia (2%), and North America (1%) [28]. These reports confirm that MRSA prevalence varies between countries and across animal species. The prevalence should be considered individually.

In Japan, the prevalence of MRSA in disease appraisal samples has been monitored since 2019 [29], resulting in the prevalence of MRSA in diseased pigs varying from 0% to 15%, with no chronological upward trend shown from 2019 to 2022. Regarding healthy livestock, a recent report suggested that the prevalence of MRSA in slaughtered pigs has increased at both the individual and the farm level over the 5-year period from 2018 to 2022 [30]. Given the potential risk of LA-MRSA transmission, monitoring changes in the prevalence of MRSA in healthy livestock is crucial. To date, however, few comprehensive and retrospective studies have reported on the prevalence of MRSA in healthy livestock across Japan.

Since the detection of LA-MRSA in a human whose family was engaged in pig farming was reported from the Netherlands in the early 2000s [31], LA-MRSA has been broadly investigated in Europe, Asia, Australia, and the United States [1,26,27]. Growing attention has likewise led to the accumulation of reports in Japan over the past 20 years. This systematic review aims to estimate the pooled prevalence of MRSA in livestock in Japan and to assess its temporal change by meta-analysis from articles published between January 2000 and March 2023.

## 2. Materials and Methods

### 2.1. Search Strategy

**Figure 1:**
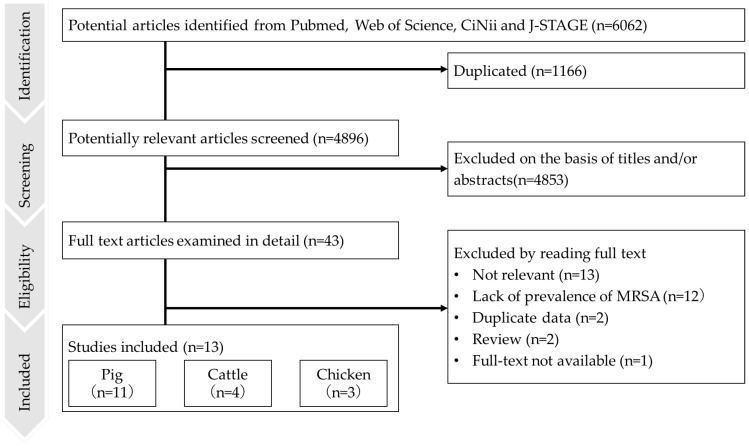
Flow diagram of the literature search and study selection according to PRISMA criteria. The sum of the number of studies in each species differs from the total number of articles included because several studies reported results for multiple species.

This systematic review was conducted according to the Preferred Reporting Items for Systematic Reviews and Meta-Analyses (PRISMA) guidelines [32], as shown in Figure 1. The study protocol was pre-registered in the Open Science Framework before starting the literature search (https://doi.org/10.17605/OSF.IO/DE2X4, accessed on 1 January 2025). On 1 April 2023, articles in English or Japanese published between January 2000 and March 2023 were searched using the following databases: PubMed, Web of Science (in English), CiNii Research (in English and Japanese), and J-STAGE (in English and Japanese). The following keywords were used for each database. Terms related to pigs (“pig/swine/piglet”), cattle (“cattle/cow/calf”), chickens (“chicken/broiler/layer”), or animals (“livestock/animals”) were combined with terms related to MRSA (“methicillin-resistant *Staphylococcus aureus*/MRSA/*Staphylococcus aureus*/*S. aureus*”) and “Japan”. Duplicate records were removed, first by Rayyan (https://www.rayyan.ai/, accessed on 1 January 2025), the initial screening tool for systematic reviews, then manually by the reviewers.

### 2.2. Inclusion and Exclusion Criteria

The titles and abstracts of the studies retrieved during the search were screened independently by two reviewers and the eligibility of the identified studies was assessed using the following inclusion and exclusion criteria. Studies that reported the prevalence of MRSA in healthy livestock or farms in Japan were included, regardless of whether they were academic or grey literature. Studies were excluded if they (i) were not focusing on domestic livestock or farms (e.g., focusing only on food, imported animals, or aquaculture), (ii) provided insufficient data to compute the prevalence of MRSA at the individual or farm level, (iii) were not written in English or Japanese, (iv) did not offer any new information (e.g., letters to the editor or reviews), or (v) were case reports of farms which were identified as positive for MRSA in advance. If two or more studies were conducted using the same samples, the study that provided the most relevant data (determined by a close reading of each study) was included in the analysis. The full text of the remaining studies was retrieved and screened for eligibility. Any disagreement between reviewers was resolved by consensus.

### 2.3. Data Extraction

Two reviewers independently extracted information from the eligible studies. The following information was collected: first author, year of publication, sampling year, sampling region, animal species, number of tested animals, number of MRSA-positive animals, number of farms tested, number of MRSA-positive farms (defined as farms with one or more MRSA-positive pigs), and, for pigs only, whether bacterial pre-enrichment was performed.

### 2.4. Data Analysis

All analyses were carried out using R version 4.2.2 and the R packages meta, metafor, and dmetar [33,34,35]. As considerable heterogeneity between studies was expected, pooled prevalences of MRSA at the individual and farm levels for each animal species were calculated using a random-effects model. The Knapp–Hartung adjustments [36] were used to calculate the 95% confidence interval (CI) around the pooled effect. A Freeman–Tukey double arcsine transformation was conducted before the calculation to stabilize variances. *I*-squared statistics (*I*^2^) were calculated to investigate the heterogeneity between studies [37]. As a criterion for evaluating *I*^2^, values of 0.25, 0.50, and 0.75 were considered to indicate small, moderate, and high levels of heterogeneity, respectively [37]. The heterogeneity variance τ-squared was calculated using the restricted maximum likelihood estimator [38].

When significant heterogeneity was observed, a subgroup analysis was conducted to identify the source of the heterogeneity [39]. In addition, a meta-regression analysis was performed to investigate the temporal trend between MRSA prevalence and sampling year as a source of heterogeneity [37]. A sensitivity analysis was performed to confirm the stability of the prevalence using the leave-one-out process, a method of removing one study and recalculating, and the Baujat plot, a method of visually identifying influential studies [40,41,42]. A funnel plot was used to visualize potential publication bias in the prevalence of MRSA at the individual pig level, and an Egger’s test was conducted to examine publication bias. For all analyses, values of *p* < 0.05 were considered statistically significant.

## 3. Results

Our literature search yielded 6062 studies, from which 14 sets of prevalence data from the 13 studies shown in Table 1 were included in the meta-analysis after screening [20,21,22,23,43,44,45,46,47,48,49,50,51]. Of those 13 studies, 11 presented MRSA prevalence data for pigs, four had data for cattle, and three had data for chickens. One study presented two sample sets for pigs across different sampling periods. Three studies provided multiple datasets for different animal species: one study presented datasets for cattle and pigs and two presented datasets for pigs, cattle, and chickens.

**Figure 2:**
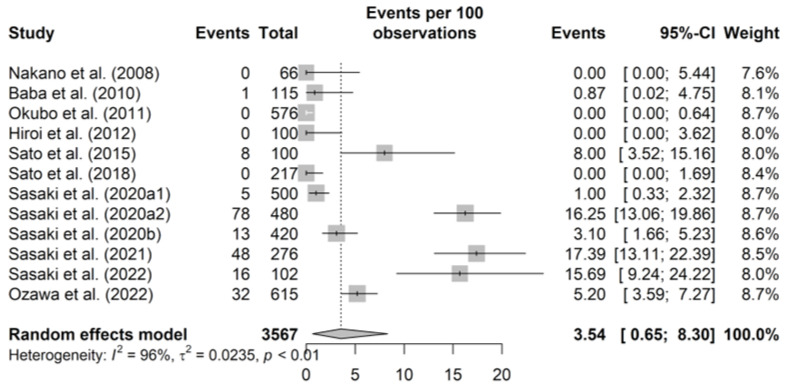
Forest plot for the prevalence of methicillin-resistant *Staphylococcus aureus* (MRSA) at the individual pig level. Pooled prevalence was achieved using the random-effects model. The squares indicate the estimated prevalences, with sizes reflecting weight and horizontal bars reflecting the 95% confidence interval (CI). “Events per 100 observations” and “events” indicate prevalence (%). The numbers after the study year distinguish multiple results in a report. The diamond indicates the summary prevalence estimate [20,21,22,23,44,45,46,47,48,49,50].

**Figure 3:**
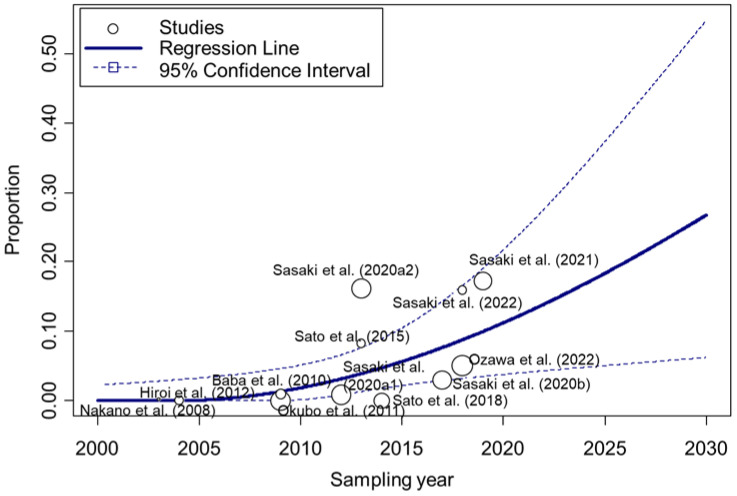
Association between prevalence of methicillin-resistant *Staphylococcus aureus* (MRSA) at the individual pig level and sampling year. The size of the circle depicts the weight of each study. The line indicates the regression line. The numbers after the study year distinguish multiple results in a report [20,21,22,23,44,45,46,47,48,49,50].

The pooled prevalence of MRSA at the individual pig level was 3.54% (95% CI 0.65–8.30, *p* for heterogeneity < 0.001, *I*^2^ = 96.4%) (Figure 2). Because of the significant heterogeneity, subgroup analyses were performed which revealed significant differences in prevalence by sampling year (0.73% for studies sampled in 2013 or before, 7.65% for studies sampled in 2014 or after; *p* = 0.020) and implementation of bacterial pre-enrichment (0% for non-pre-enrichment, 4.67% for pre-enrichment; *p* = 0.001) (Table 2). Sampling regions (prefectures) were classified into regional blocks based on a previous report [52], with “North Kanto” and “South Kanto” combined into “Kanto” and others that could not be classified into any blocks defined as ‘unclassifiable’. Regional blocks where samples were taken were also associated with prevalence of MRSA at the individual pig level (0.00%, 8.91%, 12.39%, 0.00%, and 2.07% for Hokkaido, Tohoku, Kanto, Kinki, and unclassifiable, respectively; *p* < 0.001) (Table 2). Further, a meta-regression showed that the prevalence of MRSA had increased significantly in association with the sampling year (*p* = 0.019), although significant heterogeneity remained (*I*^2^ = 94.1%, *p* < 0.001) (Figure 3, Table 3).

**Figure A1:**
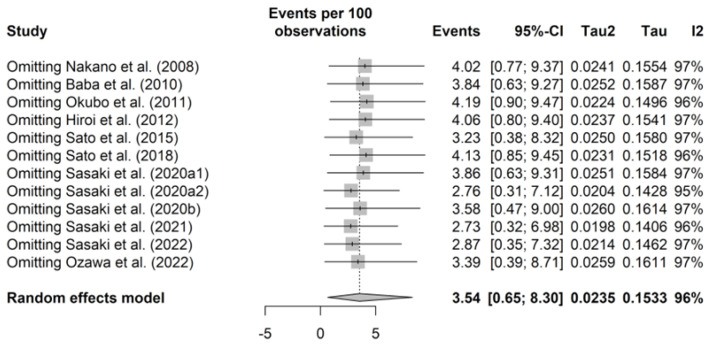
Leave-one-out analysis for individual pig-level studies. The squares indicate the estimated prevalences, with sizes reflecting the weight and horizontal bars reflecting the 95% confidence interval (CI). The diamond indicates the summary prevalence estimate. “Events per 100 observations” and “events” indicate prevalence (%). The numbers after the study year distinguish multiple results in a report [20,21,22,23,44,45,46,47,48,49,50].

**Figure A2:**
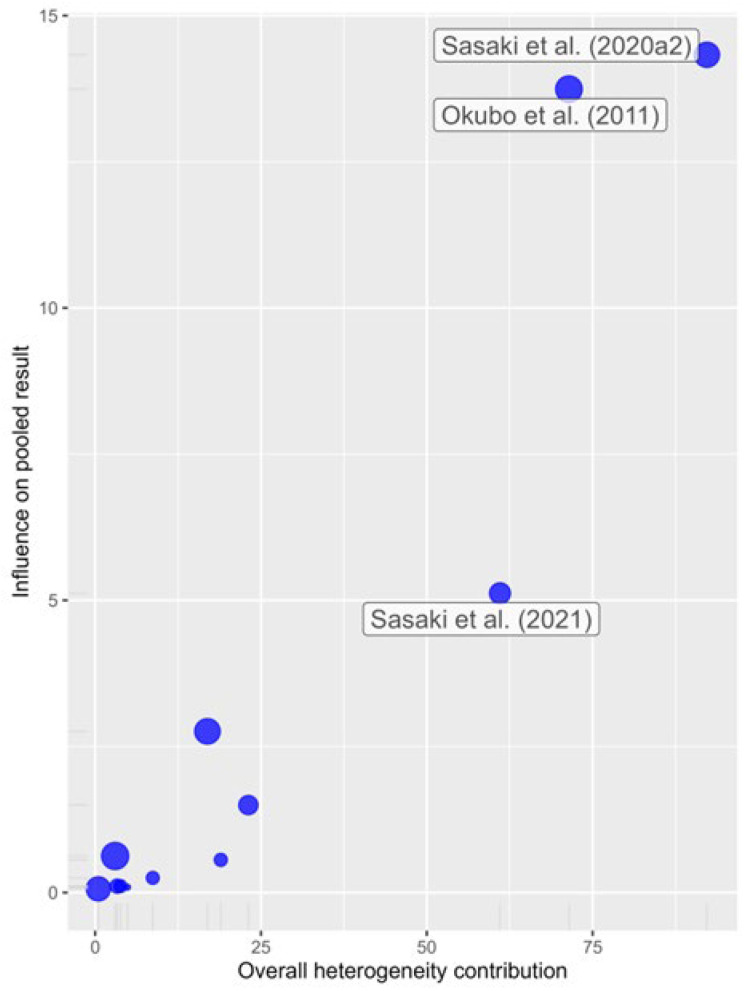
The Baujat plot showing the contribution of each study to heterogeneity and pooled prevalence of MRSA at the individual pig level. The top right points could possibly have large impacts on the overall pooled prevalence of MRSA [21,23,47].

**Figure A3:**
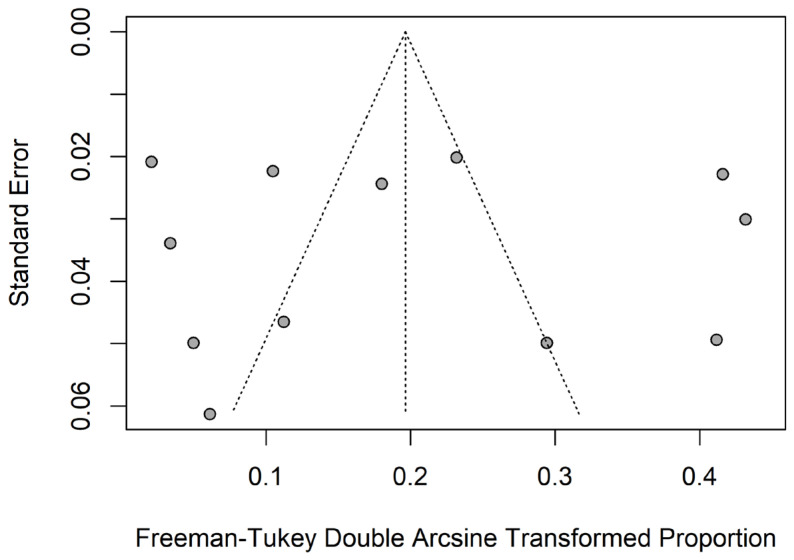
Funnel plot of the prevalence of methicillin-resistant *Staphylococcus aureus* (MRSA) at the individual pig level.

The leave-one-out analysis confirmed the prevalence of MRSA at the individual pig level, ranging from 2.73% (95% CI 0.32–6.98, *I*^2^ = 96%) to 4.19% (95% CI 0.90–9.47, *I*^2^ = 96%), indicating that the obtained prevalence was not driven by any single study (Appendix A, Figure A1). Moreover, the Baujat plot analysis revealed that three studies could possibly have had large impacts on the overall pooled prevalence of MRSA (Figure A2). Table 4 shows the prevalence and *I*^2^ after the sequential calculation excluding these studies. When the three studies were removed, the pooled prevalence was calculated as 2.35% and the heterogeneity decreased to 89.3%, but the test for heterogeneity remained significant (*p* < 0.001). The influence of the sampling year on the pooled prevalences shown in the meta-analysis remained significant if one or two of the studies considered to be outliers by the Baujat plot were excluded but became non-significant if all three studies were excluded (*p* for moderators = 0.011, 0.021, and 0.059, respectively) (Table 3). No significant publication bias was observed by the funnel plot and Egger’s test in the prevalence of MRSA at the individual pig level (*p* = 0.951) (Figure A3).

**Figure 4:**
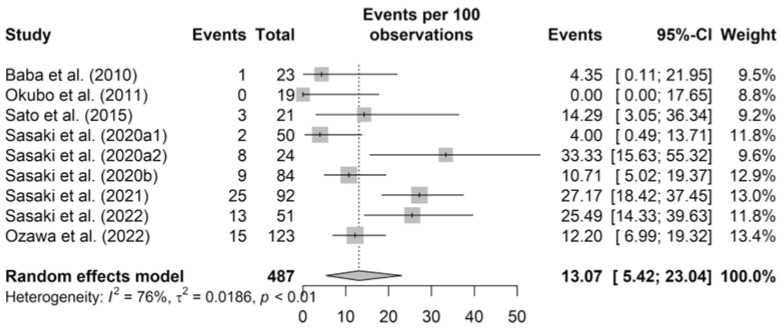
Prevalence of methicillin-resistant *Staphylococcus aureus* (MRSA) at the pig farm level. The squares indicate the estimated prevalences, with sizes reflecting the weight and horizontal bars reflecting the 95% confidence interval (CI). “Events per 100 observations” and “events” indicate prevalence (%). The numbers after the study year distinguish multiple results in a report. The diamond indicates the summary prevalence estimate [20,21,22,23,44,47,48,49].

**Figure A4:**
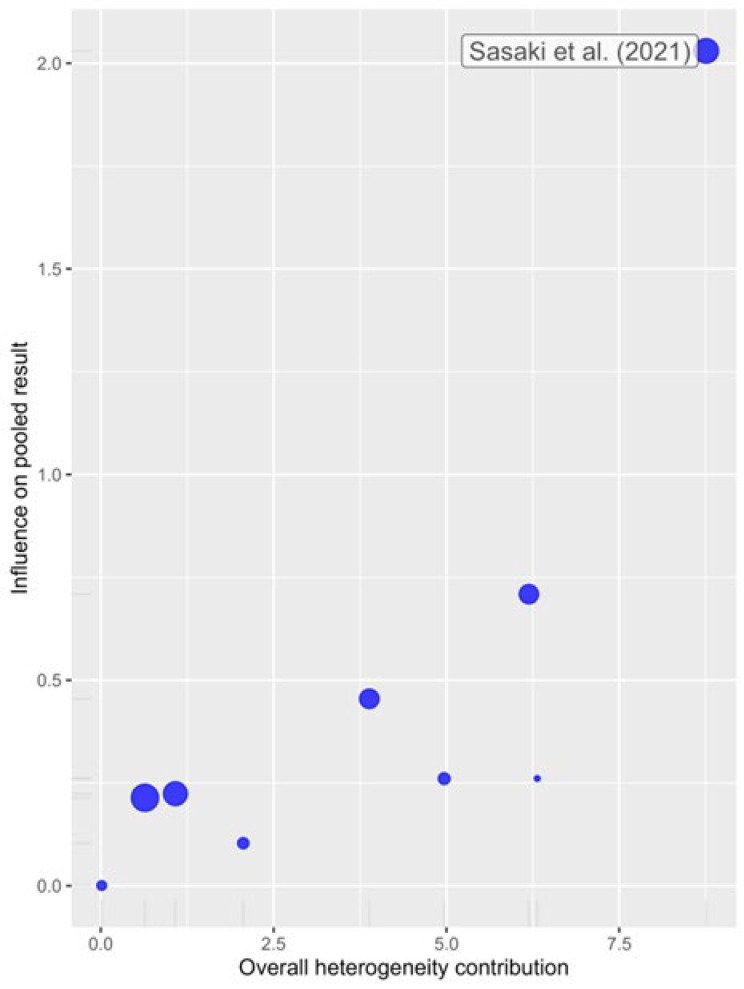
**The** Baujat plot showing the contribution of each study to heterogeneity and pooled prevalence of MRSA at the pig farm level. The top right point could possibly have a large impact on the overall pooled prevalence of MRSA [21].

The pooled prevalence of MRSA at the pig farm level was 13.07% (95% CI 5.42–23.04, *p* for heterogeneity < 0.001, *I*^2^ = 76.4%) (Figure 4). The Baujat plot revealed that one study may have influenced the overall pooled prevalence of MRSA (Figure A4). In a meta-analysis excluding that study, prevalence and *I*^2^ were 11.28% and 69.7%, respectively (Table 4).

**Figure A5:**
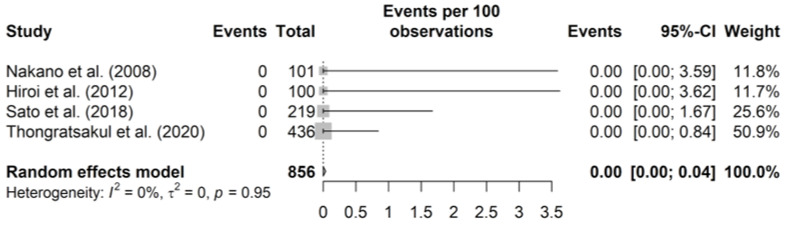
Prevalence of methicillin-resistant *Staphylococcus aureus* (MRSA) at the individual cattle level. The squares indicate the estimated prevalences, with sizes reflecting the weight and horizontal bars reflecting the 95% confidence interval (CI). The diamond indicates the summary prevalence estimate. “Events per 100 observations” and “events” indicate prevalence (%) [45,46,50,51].

**Figure A6:**
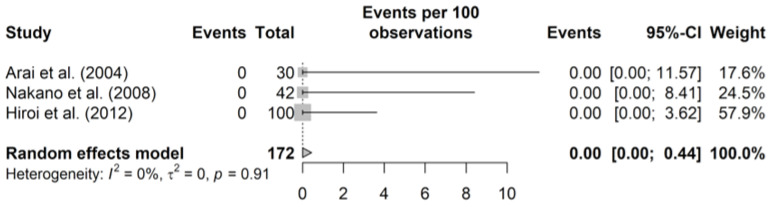
Prevalence of methicillin-resistant *Staphylococcus aureus* (MRSA) at the individual chicken level. The squares indicate the estimated prevalences, with sizes reflecting the weight and horizontal bars reflecting the 95% confidence interval (CI). The diamond indicates the summary prevalence estimate. “Events per 100 observations” and “events” indicate prevalence (%) [43,45,46].

The pooled prevalence of MRSA was 0.0% (95% CI 0.00–0.04, *p* for heterogeneity = 0.947, *I*^2^ = 0.0%) at the individual cattle level (Figure A5) and also at the individual chicken level (95% CI 0.00–0.44, *p* for heterogeneity = 0.909, *I*^2^ = 0.0%) (Figure A6). Pooled prevalences at the farm level were not calculated because only one study each reported farm-level prevalences for cattle and chickens.

## 4. Discussion

### 4.1. Main Findings

Our study resulted in a pooled MRSA prevalence of 3.54% at the individual pig level in Japan. This suggests that the prevalence of MRSA in pigs is lower in Japan than in China (11.2%) [53], Korea (7.9%) [54], the Netherlands (98.9%) [55], or Canada (4.6%) [56], but higher than in Norway (0%) [57].

Our study also found a pooled MRSA prevalence of 13.07% at the pig farm level in Japan. The prevalence of MRSA in pigs at the herd level has been reported to be 95% in Denmark, among conventional breeding pig herds [58], and 11% in Canada [56], suggesting that the prevalence of MRSA at the farm level in Japan is lower than in Denmark and higher than in Canada.

In terms of the temporal change in MRSA prevalence at the individual pig level, notably, the prevalence of MRSA at the individual pig level in Japan was significantly higher in the last decade than before. Further, the results of the meta-regression suggested the potential of a chronological upward trend since 2000, although multiple outliers could have contributed. As molecular epidemiological analyses have suggested a relationship between isolates from slaughtered pigs and imported breeding pigs [48], MRSA may have been introduced via breeding pigs imported from overseas and may have gradually spread among pigs within Japan. Given that similar cases have been reported in other countries [59,60], imported pigs might be a potential risk factor. According to the Annual Report on Animal Quarantine (https://www.maff.go.jp/aqs/tokei/toukeinen.html, accessed on 1 January 2025), breeding pigs have been continuously imported since 2002, suggesting that pigs previously imported into Japan might have been MRSA-positive and, thus, might have contributed to the current increased prevalence of MRSA. In 2024, one study revealed that the prevalence of MRSA in slaughtered pigs had increased at the individual level over the 5-year period from 2018 to 2022 [30]. Our results suggest that the change in prevalence also needs to be considered over a longer time span.

Regarding a report on factors contributing to the increased prevalence of MRSA, purchase of gilts from MRSA-positive suppliers might have been a factor contributing to the high prevalence of MRSA at the farm level [61,62,63]. However, more than half of the farms that introduced pigs from MRSA-positive farms had also been reported as MRSA-negative [64]. Additional factors might, thus, contribute to the establishment and spread of MRSA between and within farms: herd size and production type have been considered to be risk factors in fattening herds [65], while group treatment of fattening pigs with antimicrobial agents has been identified as a risk factor leading to a selective advantage for MRSA within a herd [66]. Inappropriate use of antibiotics, including long-term misuse and abuse, has been suggested to lead to widespread MRSA in pigs [67,68]. Further studies are needed to identify factors contributing to the increased prevalence of MRSA in Japan.

Our study revealed a 0% prevalence of MRSA in cattle and chickens. However, cases of mastitis in cattle caused by MRSA have been reported [69,70]. Oxacillin resistance rates in *S. aureus* isolates from disease appraisal samples were reportedly 2.4% in 2019 and 0.8% in 2020 [71]. MRSA is, therefore, considered to be established in some cattle farms in Japan. Our result of a 0% prevalence could be attributed to insufficient test power resulting from the limited number of publications included in this study.

For chickens, a couple of studies have reported identification of MRSA strains based on genotypic characteristics in chicken meat obtained from Japan [70,72]. In those studies, MRSA strains were isolated from domestic chicken meat and MRSA in chicken meat was attributed to contamination during the slaughtering process. However, the number of studies that examined prevalence with a large sample size was insufficient to draw conclusive findings. More studies with larger sample sizes are required to gain more accurate estimations.

### 4.2. Limitations

Significant heterogeneities were observed in the pooled prevalences, so caution is warranted when interpreting the data. Our results show that sampling year, sampling region, and whether pre-enrichment was performed were significantly associated with pooled MRSA prevalence at the individual pig level. However, these factors could not fully explain the heterogeneity. Other possible sources of heterogeneity may have derived from the methods. For example, several different sampling sources were used for pigs, including nasal swabs, ear-dipped liquid, body surface swabs, or fecal samples. The method of identification (colonization in MRSA-selective medium, oxacillin sensitivity, or *mecA* polymerase chain reaction) can also represent a source of heterogeneity. These two points were not analyzed because the methods employed by each study differed slightly, making group comparisons unfeasible to conduct.

Regarding outliers, which represent a possible source of heterogeneity, some controversy exists surrounding how to identify and deal with outliers in meta-analyses, and whether to exclude influential studies is not easy to determine [41,73]. As a result, we attempted to validate the robustness of our meta-analyses through the sensitivity and publication bias analyses. The results show that the pooled prevalence and the increase in the prevalence of MRSA at the individual pig level with sampling year were moderately robust. This indicates that the proportion of pigs that can represent a source of MRSA infection to humans may have been increasing in Japan, and that workers in direct contact with pigs in Japan, such as farmers and veterinarians, should pay more attention to the MRSA infection status of pigs on their farms. Given that farm workers with occupational pig exposure are considered to be at high risk of MRSA infection [74], we recommend the implementation of precautionary measures to protect farmers and veterinarians from being infected with MRSA. This might include MRSA screening tests for pigs on farms and provision of up-to-date information on MRSA to raise awareness.

## 5. Conclusions

We estimate the pooled prevalences of MRSA in livestock in Japan between 2000 and 2023. Prevalences of MRSA at the individual animal level are 3.54% for pigs, 0% for cattle, and 0% for chickens, respectively, suggesting that the MRSA prevalences are lower in Japan than in most countries. Our results also suggest that the prevalence of MRSA in pigs in Japan may have increased over the course of the last two decades. Despite some limitations, our findings strongly imply that greater attention needs to be paid to the prevention of pig-to-human MRSA transmission in Japan.

## Figures and Tables

**Table 1:** Eligible studies and their main characteristics. The numbers after the study year distinguish multiple results in a report. Two datasets named a1 and a2 were provided by Sasaki et al. [23]. Studies on pigs were classified into regional blocks.

					**Individual**		**Farm**		
**Reference**	**Sampling** **Year**	**Animal**	**Sampling** **Source**	**Sampling Region** **(Regional Blocks)**	**No. of** **Tested Animals**	**No. of** **MRSA-Positive Animals**	**No. of** **Tested Animals**	**No. of** **MRSA- Positive Animals**	**Pre-Enrichment** **(Yes/No)**
Arai et al., 2004 [43]	1997	Chicken	Nares	Fukushima	30	0	N/A	N/A	Yes
Nakano et al., 2008 [46]	2003–2005	Cattle	Nares	Hyogo and Osaka	101	0	N/A	N/A	-
		Pig	Nares	Osaka (Ki)	66	0	N/A	N/A	No
		Chicken	Nares	Hyogo	42	0	1	0	-
Baba et al., 2010 [44]	2009	Pig	Feces	The eastern part of Japan (U)	115	1	23	1	Yes
Okubo et al., 2011 [47]	2009	Pig	Nares, Carcass	Iwate, Akita, Miyagi, Tochigi, and Kanagawa (U)	576	0	19	0	Yes
Hiroi et al., 2012 [45]	2004–2006	Cattle	Nares	Unknown	100	0	N/A	N/A	-
		Pig	Nares	Unknown (U)	100	0	N/A	N/A	No
		Chicken	Skin	Unknown	100	0	N/A	N/A	-
Sato et al., 2015 [49]	2013	Pig	Nares	Ibaraki (Ka)	100	8	21	3	Yes
Sato, 2018 [50]	2014–2015	Cattle	Nares	Hokkaido	219	0	N/A	N/A	-
		Pig	Nares	Hokkaido (H)	217	0	N/A	N/A	Yes
Sasaki et al., 2020 [23]	2012–2013	Pig	Nares	Tohoku, Kanto, Tokai, Kyushu (U)	500	5	50	2	Yes
Sasaki et al., 2020 [23]	2013–2014	Pig	Nares	Kanto (Ka)	480	78	24	8	Yes
Sasaki et al., 2020 [22]	2017	Pig	Nares	Tohoku (T)	420	13	84	9	Yes
Thongratsakul et al., 2020 [51]	2016–2017	Cattle	Milk	Hokkaido	436	0	3	0	-
Sasaki et al., 2021 [21]	2019	Pig	Nares, Ears	Tohoku (T)	276	48	92	25	Yes
Ozawa et al., 2022 [48]	2018–2019	Pig	Nares	The northern to the western part of Japan (U)	615	32	123	15	Yes
Sasaki et al., 2022 [20]	2018	Pig	Ears	Tohoku, Kanto, Kyushu (U)	102	16	51	13	Yes

MRSA, methicillin-resistant *Staphylococcus aureus*; N/A, not applicable; H, Hokkaido; Ka, Kanto; Ki, Kinki; U, unclassifiable.

**Table 2:** Association of sampling year, implementation of pre-enrichment practices, and sampling regions with the prevalence of methicillin-resistant *Staphylococcus aureus* (MRSA) at the individual pig level.

			Prevalence (%)		Heterogeneity	
Subgroup		No. of Included Studies	Estimate	95% CI	*I*^2^ (%)	*p*-Value for Subgroup Differences
Sampling year	In 2013 or before	6	0.73	0.00–3.80	82.6	0.020
	In 2014 or after	6	7.65	1.09–19.04	96.5	
Pre-enrichment	Not performed	2	0.00	0.00–1.01	0.0	<0.001
	Performed	10	4.67	0.97–10.70	96.9	
Sampling region	Hokkaido	1	0.00	0.00–0.79	-	<0.001
(regional blocks)	Tohoku	2	8.91	0.00–100.00	97.6	
	Kanto	2	12.39	0.00–81.92	79.7	
	Kinki	1	0.00	0.00–2.59	-	
	Unclassifiable	6	2.07	0.00–8.71	94.4	

CI, confidence interval.

**Table 3:** Association between sampling year and prevalence of methicillin-resistant *Staphylococcus aureus* (MRSA). A sensitivity analysis was conducted by sequentially omitting studies that could possibly have large impacts on the overall pooled prevalence. The numbers after the study year distinguish multiple results in a report.

	Coefficient	Intercept	*p*-Value for Moderators	*I*^2^ (%)	*p*-Value for Residual Heterogeneity
For meta-regression of the prevalence of MRSA at individual pig level					
Overall	0.0200	−40.1395	0.019	94.11	<0.001
Without Sasaki et al. [23]	0.0198	−39.6762	0.011	91.07	<0.001
Without Sasaki et al. [23] and Okubo et al. [47]	0.0185	−37.0228	0.021	90.72	<0.001
Without Sasaki et al. [23], Okubo et al. [47], and Sasaki et al. [21]	0.0147	−29.3438	0.059	88.28	<0.001

**Table 4:** Sensitivity analysis to examine the pooled prevalence of methicillin-resistant *Staphylococcus aureus* (MRSA) at both the individual pig and the pig farm level, sequentially omitting studies that could possibly have large impacts on the overall pooled prevalence.

			Prevalence (%)		Heterogeneity	
	No. of Included Studies (Datasets)	Total no. of Tested Animals	Estimate	95% CI	*I*^2^ (%)	*p*-Value for Between-Study Heterogeneity
For the prevalence of MRSA at the individual pig level						
Without Sasaki et al. [23]	11 (11)	3087	2.76	0.31–7.12	95.0	<0.001
Without Sasaki et al. [23] and Okubo et al. [47]	10 (10)	2511	3.34	0.46–8.27	93.8	<0.001
Without Sasaki et al. [23], Okubo et al. [47], andSasaki et al. [21]	9 (9)	2235	2.35	0.18–6.34	89.3	<0.001
For the prevalence of MRSA at the pig farm level						
Without Sasaki et al. [21]	7 (8)	395	11.28	3.81–21.50	69.7	0.002

The numbers after the study year distinguish multiple results in a report. CI, confidence interval.

## Data Availability

All data are available on request from the first author (hanamoto-sayoko0908@g.ecc.u-tokyo.ac.jp).
